# Modulation of Mismatch Repair and the SOCS1/p53 Axis by microRNA-155 in the Colon of Patients with Primary Sclerosing Cholangitis

**DOI:** 10.3390/ijms23094905

**Published:** 2022-04-28

**Authors:** Monika Adamowicz, Iga Stukan, Piotr Milkiewicz, Andrzej Bialek, Malgorzata Milkiewicz, Agnieszka Kempinska-Podhorodecka

**Affiliations:** 1 Department of Medical Biology, Pomeranian Medical University in Szczecin, 70-111 Szczecin, Poland; monikadamowicz@gmail.com (M.A.); iga.stukan@pum.edu.pl (I.S.); malgorzata.milkiewicz@pum.edu.pl (M.M.); 2 Liver and Internal Medicine Unit, Medical University of Warsaw, 02-097 Warsaw, Poland; p.milkiewicz@wp.pl; 3 Translational Medicine Group, Pomeranian Medical University, 70-111 Szczecin, Poland; 4 Department of Gastroenterology, Pomeranian Medical University in Szczecin, 70-111 Szczecin, Poland; bialekab@pum.edu.pl

**Keywords:** miR-155, biomarker, microsatellite instability, inflammation, primary sclerosing cholangitis, colorectal cancer

## Abstract

Deficient mismatch repair (MMR) proteins may lead to DNA damage and microsatellite instability. Primary sclerosing cholangitis (PSC) is a risk factor for colitis-associated colon cancer. MiR-155 is suggested to act as a key regulating node, linking inflammation and tumorigenesis. However, its involvement in the chronic colitis of PSC-UC patients has not been examined. We investigated the involvement of miR-155 in the dysregulation of MMR genes and colitis in PSC patients. Colon tissue biopsies were obtained from patients with PSC, PSC with concomitant ulcerative colitis (PSC-UC), uncomplicated UC, and healthy controls (*n* = 10 per group). In the ascending colon of PSC and PSC-UC patients, upregulated miR-155 promoted high microsatellite instability and induced signal transducer and activator of transcription 3 (STAT-3) expression via the inhibition of suppressors of cytokine signalling 1 (SOCS1). In contrast, the absence of miR-155 overexpression in the sigmoid colon of PSC-UC patients activated the Il-6/S1PR1 signalling pathway and imbalanced the IL17/FOXP3 ratio, which reinforces chronic colitis. Functional studies on human intestinal epithelial cells (HT-29 and NCM460D) confirmed the role of miR-155 over-expression in the inhibition of MMR genes and the modulation of p53. Moreover, those cells produced more TNFα upon a lipopolysaccharide challenge, which led to the suppression of miR-155. Additionally, exposure to bile acids induced upregulation of miR-155 in Caco-2 cell lines. Thus, under different conditions, miR-155 is involved in either neoplastic transformation in the ascending colon or chronic colitis in the sigmoid colon of patients with PSC. New insight into local modulation of microRNAs, that may alter the course of the disease, could be used for further research on potential therapeutic applications.

## 1. Introduction

Primary sclerosing cholangitis (PSC) is a chronic biliary disorder with a complex aetiology, characterised by the progressive destruction of the biliary tract (and consequently the liver), through the mechanisms of autoimmunity and cholestasis. PSC primarily affects men and is commonly accompanied by inflammatory bowel disease (IBD), predominantly in the form of ulcerative colitis (UC) [1]. Accumulating evidence has suggested that the presence of PSC is an important additional risk factor for the development of colorectal neoplasia in patients with UC (PSC-UC) [2]. This risk is thought to be 4–10-times greater than the risk of developing colorectal carcinoma (CRC) in patients with UC with PSC, and develops at a much younger age than in patients with UC alone [3]. This may indicate differences in the pathogenesis of inflammation and CRC in PSC-UC patients in comparison to patients with UC alone. Broomé et al. [4] first proposed an association between PSC and CRC in UC patients in 1992; however, currently, there is no molecular explanation for how PSC increases the risk of CRC.

MicroRNA (small non-coding RNA molecules) are key players in the regulation of immune response and their expressions are dysregulated in different types of cancer. A plethora of miRNAs has been implicated in various liver diseases. Based on our initial results from microarray analysis (commercially performed by Microarray Core facility at Boston University), we chose miR-155, since its expression was significantly increased in samples from our cohort of PSC patients, and this microRNA (miRNA) is known as a multifunctional regulator of naïve and adaptive immunity.

Moreover, miR-155 can regulate mismatch repair (MMR) genes’ (e.g., MLH1, MSH2, MSH6) expression to influence genomic stability in CRC [5]. The expression patterns of MMR proteins are one parameter by which colorectal cancers can be classified [6,7,8]. MMR proteins are nuclear enzymes that form heterodimers that bind to areas of abnormal DNA and initiate its removal. Loss of MMR proteins leads to an accumulation of DNA replication errors, which is termed microsatellite instability (MSI) [8].

MiR-155 is most commonly upregulated in malignancy and is hypothesised to target tumour suppressor genes, such as the suppressor of cytokine signalling 1 (SOCS1). Furthermore, SOCS1 contributes to p53 activation and regulates the process of oncogene-induced senescence, which is an important tumour suppressor response [9].

Our previous research showed that miR-155 not only directly inhibits SOCS1 expression, but also increases the production of pro-inflammatory mediators in the liver of PSC patients [10]. In fact, SOCS1 can promote or inhibit the activation of macrophages and dendritic cells, and participate in T cell differentiation and innate and adaptive immune regulation [11,12].

Moreover, miR-155 is able to potentiate the inflammation response, and its expression is upregulated upon lymphocyte activation to control cell proliferation and differentiation [13]. However, the involvement of miR-155 in chronic inflammation in the colons of PSC-UC patients has not been investigated. Sphingosine-1-phosphate receptor 1 (S1PR1) was found to be a new target gene of miR-155 by in vitro and in vivo studies [14]. Degradation of S1PR1 prevents lymphocytes from sensing the sphingosine-1-phosphate (S1P) gradient from the lymphoid organs to the blood, thus, blocking egress and inducing lymphopaenia [15]. Furthermore, pro-inflammatory cytokines (including IL-6) raise the level of signal transducers and activators of transcription 3 (STAT3), which, in turn, induces a higher expression of S1PR1 [16]. The role of S1PR1 in tumour formation is consistent with the finding of S1PR1 upregulation in STAT3-positive tumours and in oestrogen receptor-positive breast cancer cells. This has been further confirmed by experiments showing that prevention of S1PR1 activation inhibits tumour growth, metastasis and persistent STAT3 activation [17]. The tight control of S1PR1 expression is crucial for cell homeostasis.

Depending on its target genes, miR-155 is able to potentiate pro-inflammatory responses [18,19] or oncogenic activity [20,21] in the colon. Therefore, in this study, we aimed to confirm the interaction between miR-155 and its targets (including MMR genes, SOCS1, and S1PR1), given that their molecular mechanisms have not yet been elucidated in patients with PSC. Additionally, given that miR-155 regulates the Th17/Treg ratio by targeting SOCS1 [22], and that miR-155 and STAT3 form an axis that promotes the expansion of pathogenic Th17 cells [23], we investigated the contribution of miR-155 in the modulation of the immune response in colonic tissues and peripheral blood mononuclear cells (PBMCs) of PSC patients, with and without concurrent UC. Besides, these molecular analyses of human samples were complemented by functional studies in three human intestinal cell lines (Caco-2, HT-29, and NCM460D).

## 2. Results

**Figure 1:**
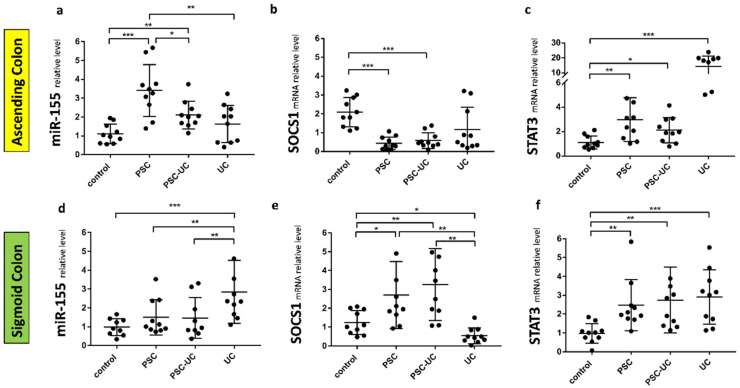
Expression of miR-155, suppressor of cytokine signalling 1 (SOCS1), and signal transducer and activator of transcription 3 (STAT3) mRNAs in human colon tissue. A scatter dot plot showing the relative expression levels of miR-155 (**a**,**d**), SOCS1 (**b**,**e**), (STAT3) (**c**,**f**) in both the ascending (**a**–**c**) and sigmoid colons (**d**–**f**) of controls, primary sclerosing cholangitis patients (PSC), PSC and concomitant ulcerative colitis patients (PSC-UC), and ulcerative colitis (UC). Levels of gene expression were normalised to an endogenous reference, miR-191 for miRNA or 18S RNA for other genes, and presented as a fold-change relative to healthy controls. Results are representative of 10 independent experiments per group. Dots illustrate each patient, and data are presented as mean plus interquartile range (IQR) * *p*-value < 0.05, ** *p*-value < 0.01, *** *p*-value < 0.001.

In the ascending colon, miR-155 expression was substantially increased in PSC and PSC-UC patients (3.4 ± 0.4 vs. 1.1 ± 0.2 in controls, *p* = 0.0001, and 2.1 ± 0.2 vs. 1.1 ± 0.2 in controls, *p* = 0.002, respectively, Figure 1a), which was associated with considerable suppression of SOCS1 mRNA (0.4 ± 0.3 vs. 1.9 ± 0.9 in controls, *p* = 0.0002, and 0.6 ± 0.4 vs. 1.9 ± 0.9 in controls, *p* = 0.0005; respectively, Figure 1b). The upregulation of miR-155 was higher in PSC compared to PSC-UC patients (*p* = 0.02). In contrast, miR-155 and SOCS1 mRNA levels were unchanged in UC patients in comparison to controls (Figure 1a,b). In the ascending colon, STAT3 expression was enhanced in all of the patient groups, i.e., PSC, PSC-UC and UC (Figure 1c). We observed a negative correlation between miR-155 and SOCS1 mRNA (r = −0.4, *p* = 0.03) or STAT3 (r = −0.8, *p* = 0.002). We suggest that miR-155 is involved in the pathogenesis of cancer tumorgenesis in the ascending colon of PSC patients.

In the sigmoid colon of PSC patients with and without UC, miR-155 levels were similar to control values (Figure 1d), whereas the SOCS1 mRNA levels were substantially increased (*p* = 0.004 and, *p* = 0.03 respectively, Figure 1e). In UC patients, miR-155 was induced (*p* = 0.0004 vs. controls; *p* = 0.008 vs. PSC, and *p* = 0.006 vs. PSC-UC) and was accompanied by the suppression of SOCS1 (*p* = 0.02 vs. controls, *p* = 0.002 vs. PSC, and *p* = 0.004 vs. PSC-UC). Moreover, in the sigmoid colon of all patients, a substantial upregulation of STAT3 expression was observed (*p* = 0.004 in PSC vs. controls; *p* = 0.0067 in PSC-UC vs. controls, and *p* = 0.0009 in UC vs. controls) (Figure 1f). This may suggest differences in inflammation in the sigmoid colon of PSC-UC patients in comparison to patients with UC alone.

**Figure 2:**
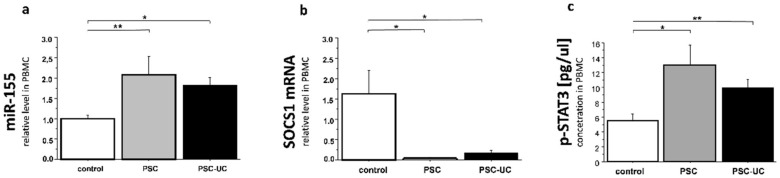
Relative expression of miR-155, SOCS1, and the concentration of p-STAT3 protein in PBMCs. Peripheral blood mononuclear cells were obtained from PSC and PSC-UC patients, and healthy subjects. Significant upregulation of miR-155 in the PSC and PSC-UC groups was accompanied by the inhibition of SOCS1 and enhancement of p-STAT3. Bars indicate the mean ± SEM * *p*-value < 0.05, ** *p*-value < 0.01.

Appreciating the role of miR-155 in the modulation of immunity and to further address its potential involvement in colitis, we evaluated the expression of miR-155 and its downstream targets in PBMCs. The expression of miR-155 in PBMCs was significantly increased in PSC and PSC-UC patients (2-fold, *p* = 0.01 vs. controls, and 1.8-fold, *p* = 0.04 vs. controls, respectively; Figure 2a). In PSC patients with and without concurrent UC, the level of SOCS1 mRNA was significantly lower (over 70% reduction, *p* = 0.05 in PSC-UC vs. controls, and over 80% reduction, *p* = 0.02 in PSC vs. controls, Figure 2b). We further evaluated the concentration of phospho-STAT3 (p-STAT3) in the PBMCs of PSC patients (Figure 2c) and observed significantly upregulated p-STAT3 expression in both PSC and PSC-UC patients (13.0 ± 2.6 pg/µL vs. 5.5 ± 0.9 in controls, *p* = 0.002, and 9.8 ± 1.2 pg/µL vs. 5.5 ± 0.9 in controls, *p* = 0.01, respectively).

**Figure 3:**
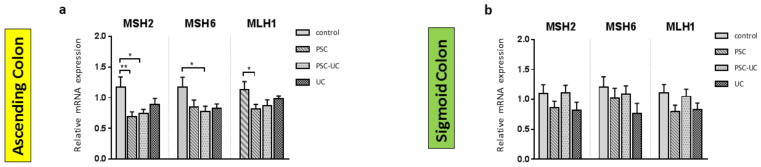
The expression patterns of mismatch repair (MMR) components in colonic tissue. Levels of gene expression were normalised to 18S RNA and presented as a fold-change relative to healthy controls. Bars indicate the mean ± SEM * *p*-value < 0.05, ** *p*-value < 0.01.

As MiR-155 is associated with the modulation of MMR repair machinery, we examined the expression of MSH2, MSH6 and MLH1 in colonic tissue. In the ascending colon of PSC patients with and without UC, the expression of MSH2 mRNA was downregulated (0.6 ± 0.1 vs. 1.2 ± 0.2 in controls, *p* = 0.005; and 0.7 ± 0.1 vs. 1.2 ± 0.2 in controls, *p* = 0.01, respectively; Figure 3a). The inhibition of MSH6 expression was observed in the ascending colon of PSC patients with concurrent UC (0.7 ± 0.3 vs. 1.2 ± 0.2 in controls, *p* = 0.01), whereas downregulation of MLH1 mRNA was observed in the ascending colon of PSC patients (0.8 ± 0.1 vs. 1.1 ± 0.1 in controls, *p* = 0.02). However, in the ascending colon of UC patients, and in the sigmoid colon of all patients, the levels of MSH2, MSH6, and MLH were unchanged (Figure 3a,b).

**Figure 4:**
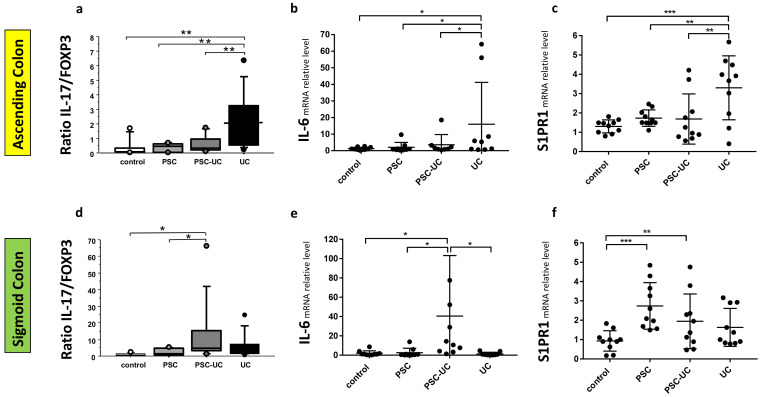
Distinct intestinal IL-17/FOXP3 ratio and the expression of IL-6 and S1PR1 mRNAs in the colonic tissue of PSC patients with and without UC. * Scatter dot plot showing IL-17 and FOXP3 proportions (**a**,**d**), and the relative expression levels of IL-6 (**b**,**e**) and S1PR1 (**c**,**f**) in the ascending (**a**–**c**) and sigmoid colons (**d**–**f**) of controls, PSC, PSC with UC, and UC patients. Levels of gene expression presented as fold-change relative to healthy controls were normalised to the endogenous reference 18S RNA. Results are representative of 10 independent experiments per group. Dots illustrate each patient and data are presented as means plus interquartile ranges (IQRs) * *p*-value < 0.05, ** *p*-value < 0.01, *** *p*-value < 0.001.

As miR-155 is reported to be modulated by Treg-specific factors, such as forkhead box O3 (FOXP3), we evaluated the mRNA level of this gene in colonic tissue. In addition, the expression of IL-17, the best marker for immuno-phenotyping of Th17 cells, was evaluated. There was an increased level of IL-17 mRNA (27-fold increase vs. controls, *p* = 0.04, data not shown) and an imbalanced IL-17/FOXP3 ratio in the sigmoid colon tissue of patients with PSC-UC (*p* = 0.02 vs. control and *p* = 0.04 vs. PSC; Figure 4d). Similarly, an imbalance in the IL-17/FOXP3 ratio was observed in the ascending colon of patients with UC (*p* = 0.004 vs. controls; *p* = 0.008 vs. PSC-UC; *p* = 0.006 vs. PSC; Figure 4a). The IL-17/FOXP3 ratio imbalance was associated with a very high expression of pro-inflammatory IL-6. In the sigmoid colon of PSC-UC patients, the expression of IL-6 was increased over 40-fold (*p* = 0.04 vs. controls, and *p* = 0.01 vs. PSC, and *p* = 0.01 vs. UC, Figure 4e), while in the ascending colon of UC patients, we observed a 13-fold increase in IL-6 (*p* = 0.02 vs. controls; *p* = 0.03 vs. PSC, and *p* = 0.05 vs. PSC-UC, Figure 4b). Based on the observed IL-6 enhancement, we examined the expression of the receptor S1PR1, which is known to be modulated by IL-6. In the ascending colon of PSC and PSC-UC patients, the S1PR1 mRNA levels remained at control values, while in UC patients, the expression of S1PR1 mRNA was considerably enhanced (*p* = 0.0001, Figure 4c). This substantial increase was also evident in comparison to PSC and PSC-UC patients (*p* = 0.003 and *p* = 0.002, respectively). We found a negative correlation between S1PR1 mRNA and miR-155 (r = −0.5, *p* = 0.009). However, the opposite was observed in the sigmoid colon of PSC and PSC-UC patients, given that the expression of S1PR1 mRNA was enhanced (*p* = 0.0005, and *p* = 0.004, respectively), whereas in UC patients, it remained at control values (Figure 4f). The expression of S1PR1 negatively correlated with miR-155 (r = −0.4, *p* = 0.04) but positively with STAT3 (r = 0.4, *p* = 0.04).

**Figure 5:**
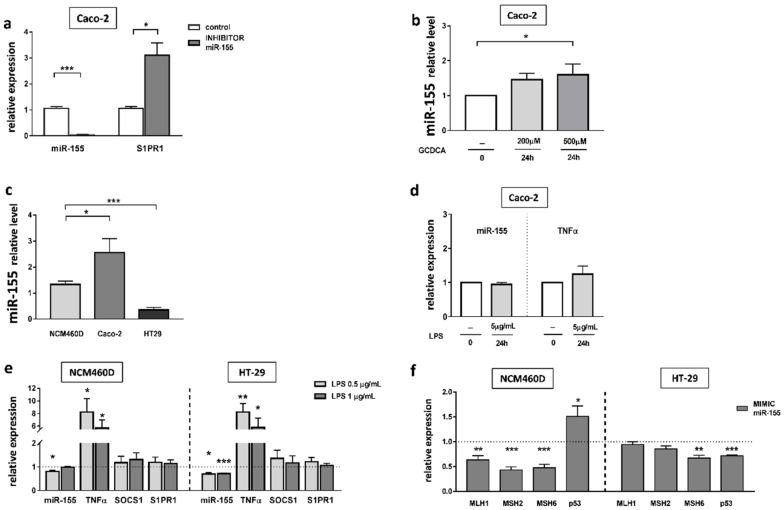
The effect of miRNA inhibition on miR-155 and S1PR1 expression (**a**). Dose-dependent effect of GCDCA on miR-155 expression (**b**). The basal miR-155 expression in colonic cell lines (**c**). miR-155 and TNF α mRNA levels in Caco-2 after lipopolysaccharide (LPS) exposure (**d**). The expression of miR-155, TNFα, SOCS1 and S1PR1 in HT29 and NCM460D cells following 24 h treatment with LPS (**e**). Modulation of MMR (MLH1, MSH2, MSH6) and p53 gene expression levels after miR-155 mimic transfection in colonic cell lines (**f**). At least three independent experiments were conducted. The dotted lines represent control values. Levels of gene expression were normalised to the endogenous reference miR-191/miR-16 for miRNA, or 18S RNA for other genes. Bars indicate the mean ± SEM * *p*-value < 0.05, ** *p*-value < 0.01, *** *p*-value < 0.001.

In previous work, we conducted a set of in vitro functional studies on the human intestinal epithelial cells Caco-2 [24], and showed an inverse relationship between miR-155 and SOCS1. Therefore, in this study, we examined another target for miR-155, the receptor S1PR1. We investigated whether miR-155 is directly involved in the regulation of S1PR1 gene expression. Transfection of the inhibitor targeting miR-155 into cells effectively suppressed miR-155 and simultaneously induced S1PR1 mRNA expression (3.1 ± 0.5 vs. 1.1 ± 0.2 in controls, *p* = 0.01; Figure 5a). We further verified the relationship between the expression of miR-155 and glycochenodeoxycholic acid (GCDCA) in the Caco-2 cell line. The expression of miR-155 was substantially increased following exposure for 24 h to 500 µM GCDCA (1.5-fold, *p* = 0.05 vs. controls; Figure 5b).

In normal cells derived from colonic mucosa (NCM460D) and cancerous cell lines (Caco-2 and HT-29), miR-155 expression displayed an altered profile at baseline (Figure 5c). We found that the miR-155 level varied between cancerous cell lines in relation to NCM460D cells. In HT-29 cells, the expression of miR-155 was decreased (3.8-fold vs. NCM460D cells, *p* < 0.0001), whereas in Caco-2 cells, it was increased (2.2-fold vs. NCM460D cells, *p* = 0.02). To gain insight into the effects of miR-155 during ongoing inflammation in the intestine, the expression of miR-155 was measured following LPS exposure in Caco-2 cells (Figure 5d). The results uncovered no significant difference in miR-155 levels and no changes were found in the major pro-inflammatory cytokine, TNF-α. Some sources have reported a lack of responses to LPS in Caco-2 cells, attributed to a low expression of TLR4 receptors on these cells [25]. Therefore, we conducted further experiments on HT-29 and NCM460D cells lines. Since the literature varies regarding LPS concentrations used in these particular cell lines, we decided to use two concentrations, namely 0.5 µg/mL and 1 µg/mL of LPS (Figure 5e). Both HT-29 and NCM460D cells responded to these concentrations. The elevated expression of TNFα was detected in HT-29 (8.3-fold increase, *p* = 0.002 and 5.8-fold increase, *p* = 0.03, respectively, for both doses of LPS) and in NCM460D (8.2-fold increase, *p* = 0.01 and 5.7-fold, *p* = 0.02, respectively, for both doses of LPS). We found that upon stimulation with LPS, miR-155 expression was inhibited in HT-29 cells at both concentrations (0.7 ± 0.1 at 0.5 µg/mL LPS, *p* = 0.002 vs. controls, and 0.7 ± 0.04 at 1 µg/mL LPS, *p* < 0.0001 vs. controls), and in NCM460D cells after treatment with 0.5 µg/mL LPS (0.8 ± 0.05, *p* = 0.005 vs. controls).

To confirm the relationship between MMR genes and miR-155, we transiently transfected NCM460D and HT-29 cells with an miR-155 mimic (Figure 5f). Results from the NCM460D cell line confirmed that, in normal non-tumorigenic cells, the response to miR155 led to a significant downregulation of MLH1 (40% reduction, *p* = 0.005), MSH2 (60% reduction, *p* = 0.0001), and MSH6 (50% reduction, *p* = 0.0003) in comparison to non-transfected cells. The drops were accompanied by an enhanced expression of p53 (1.5-fold increase, *p* = 0.03 vs. non-transfected cells). In the case of HT-29 cells, we observed a downregulation of only one of the MMR genes i.e., MSH6 (30% reduction, *p* = 0.004 vs. controls), and a reduction in p53 mRNA (30% reduction, *p* = 0.0001 vs. controls).

## 3. Discussion

One of the most prominent microRNAs implicated in both inflammation and cancer is miR-155. The importance of miR-155 deregulation in colitis arises from its cellular role, as either an inhibitor of oncogenic suppressors or a modulator of anti-inflammatory pathways [26]. Our results suggest that, first, miR-155 may promote carcinogenesis via the modulation of the SOCS1/p53 axis and high microsatellite instability (MSI-H), as it was observed in the ascending colon of PSC patients. Secondly, in the absence of miR-155 over-expression, the activated IL-6/S1PR1 signalling pathway reinforced chronic colitis in the sigmoid colon of PSC-UC patients. The biological relevance of these findings was confirmed in human intestinal cell lines in vitro.

The expression of miR-155 was substantially increased in the ascending colon of PSC patients with or without UC, which was in contrast to UC patients for whom miR-155 was significantly enhanced only in the sigmoid colon. Interestingly, in patients with PSC, the increased levels of miR-155 were seen in those parts of the colon where high concentrations of secondary bile acids (BAs) were observed. It has been established that prolonged exposure to high levels of BAs can lead to the generation of genomic instability, the development of apoptosis resistance and, ultimately, cancer [27]. The effect of toxic GCDCA on miR-155 expression was further confirmed in the Caco-2 cell line.

Our study also demonstrated that increased miR-155 expression is associated with the downregulation of SOCS1 in the ascending colon of PSC patients, with and without UC, and in the sigmoid colon of UC patients. The direct effect of miR-155 on SOCS1 expression was confirmed in our earlier studies, which showed a substantial inhibition of SOCS1 mRNA levels, in response to enhanced activation of miR-155 [24]. MiR-155, via suppression of SOCS1, exaggerates immune responses in the colonic mucosa of patients with IBD [28,29,30,31]. In UC, the inflammatory process starts in the rectum and progresses up to the sigmoid colon, and the descending and transverse colons [32]. In colitis that co-exists with PSC, colonic inflammation usually affects the right colon, i.e., it starts from the ascending colon and progresses in the opposite direction. Pro-inflammatory mediators, such as TNF-α, have been shown to be a positive modulator of miR-155, which affects miR-155 production in macrophages [33]. The elevated level of miR-155 correlates with the upregulation of TNF-α and the downregulation of SOCS1 in rheumatoid arthritis [34,35]. In our previous study, we observed the upregulation of TNF-α mRNA in the ascending colon of PSC-UC patients and in the sigmoid colon of UC patients [36]. Of note, in this study, we demonstrated the increased expression of miR-155 in the same parts of the colon. In accordance with our findings on human biopsies, TNF-α was shown to contribute to the induction of miR-155 [37]. The persistently increased levels of TNF-α can drive miR-155 overexpression, and consequently, lead to the inhibition of SOCS1. MiR-155 activates STAT3 by targeting SOCS1 [38], and it is worth emphasising that STAT3 activation can occur before histological changes in the intestinal tissue are detectable [22]. Our study demonstrated correlations between miR-155, SOCS1, and STAT3 in the ascending colon of PSC patients, with or without concurrent colitis, and in the sigmoid colon of UC patients. Similarly, in the PBMCs of PSC patients, the upregulation of miR-155 was associated with the suppression of SOCS1 and the enhanced expression of phospho-STAT3. Phosphorylated STATs can trigger the expression of genes involved in inflammatory responses [39] and angiogenesis [40], and STAT3 is associated with cancer pathogenesis [41].

MSI-H tumours occur when two or more of the five markers (i.e., MLH1, MSH2, MSH6, PMS1, or PMS2) show instability [42]. These tumours are predominantly found in the proximal colon and comprise approximately 15% of colorectal cancers. The finding of MSI-H in CRC has been associated with a favourable patient prognosis [43,44] but, paradoxically, a poor response to 5-fluorouracil-based chemotherapy [45,46,47]. This study, to our knowledge, for the first time, analysed MSI in the colon of PSC patients and demonstrated that the overexpression of miR-155 in the ascending colon was inversely related to the level of MSH2, MSH6, and MLH1 mRNA, whereas the expression of MMR mRNA was changed in the colon of UC patients. Our data are in line with other reports showing that MSI was not observed in stromal cells at any sites, including those within colitis cancer/dysplasia lesions in UC patients [48,49]. Thus, the microsatellite alterations in the ascending colon are a factor that clearly differentiates patients with UC alone from patients with PSC-UC.

We previously observed a substantial suppression of the p53 gene in the ascending colon of PSC patients, which might predispose them to pro-tumorigenic transformations [50]. A mutation in the p53 gene occurs in 34% of proximal colon tumours [51]. In contrast, in the sigmoid colon of UC patients, the levels of p53 were unchanged in comparison to controls [50]. This is in line with reports showing that approximately half of UC-associated tumours do not have p53 alterations [52,53,54]. This is related to the fact that carcinomas occurring in UC do not arise *de novo* but are preceded by, and evolve from, dysplastic mucosa, which is analogous to the development of sporadic colorectal carcinomas from adenomas [53,55]. Our in vitro analysis on the effect of miR-155 overexpression demonstrated a cell-type dependent response. In healthy epithelial NCM460D cells, miR-155 upregulation inhibited MLH1, MSH2, and MSH6 expression, which was associated with the induction of the p53 gene. In the context of DNA damage, the enhanced expression of p53 initiated various pathways, including cell cycle arrest, DNA repair, senescence, and apoptosis [56]. However, in adenocarcinoma HT-29 cells, the contrasting effect was observed as only MSH6 being suppressed, and the p53 gene was significantly downregulated. This suggests that miR-155 more efficiently suppresses the p53 gene in cancer cells than in healthy ones.

The second important observation from this study is related to the IL-6/STAT3/S1PR1 axis. Interleukin IL-6, together with TGF-beta, induces the development of Th17 cells from naïve T cells [57] and also inhibits TGF-beta-induced Treg differentiation [58,59]. At mucosal sites, IL-6 promotes Th17 development [60] and has an important role in regulating the balance between Th17/Treg in IBD [61]. The disturbed Th17/Treg ratio [22] is associated with various autoimmune and inflammatory diseases [62,63,64,65,66]. IL-17A is produced by Th17 cells and binds to receptors on the basal surface of intestinal epithelial cells to produce antimicrobial molecules that limit bacterial penetration through the epithelial barrier [67]. Human Th17 cells directly secrete inflammatory cytokines, such as IL-8, IFN-γ, TNF, and GM-CSF [55]. In turn, Treg cells control effector T cells, maintain tolerance against self or foreign antigens, and are essential for suppressing IBD [68,69]. Recent studies have suggested that miR-155 controls the properties of Treg, and the expression of miR-155 is itself controlled by Treg-specific factors, such as FOXP3 [70]. In humans and mice, the mutations in the FOXP3 locus on the X chromosome are associated with severe autoimmunity [71,72,73]. In this study, the significant imbalance in the IL-17/FOXP3 ratio, which was accompanied by IL-6 upregulation, was observed in both the sigmoid colon of PSC-UC patients and the ascending colon of UC patients, indicating persistent inflammation in these parts of the colon.

In response to inflammatory, environmental stimuli, IL-6 raises the level of STAT3, which, in turn, induces higher expression of S1PR1 [74]. S1PR1 stimulates the STAT3 protein through a positive feedback mechanism, causing the level of this protein to remain elevated, which leads to more severe inflammatory processes [75]. In our study, the significant differences in S1PR1 mRNA levels were apparent between the groups, and S1PR1 mRNA expression was negatively correlated with miR-155 expression. Thus, S1PR1 mRNA levels were upregulated in the sigmoid colon of PSC-UC patients and in the ascending colon of UC patients, where miR-155 was not enhanced. The observed negative correlation between miR-155 and S1PR1 mRNA was further confirmed in intestinal epithelial cell lines. Our observations concerning the involvement of S1PR1 in Th17 development are supported by Garriss et al. [76]. Our results suggest that enhanced production of IL-6, associated with the upregulation of S1PR1, is a critical effector in chronic intestinal inflammation, which may result in neoplastic transformation.

Despite the strengths of this study, some limitations should be noted. First of all, the number of analysed samples per each group was only 10 and, for sure, if it was higher, then the results would be more substantiated. However, PSC is a rare liver condition that means only a scarcity of biological material was available to us. An additional limitation may be the older age of controls when compared to studied groups. Nevertheless, it does not reach a statistical significance when controls are compared to the UC or PSC-UC group. We believe that the age difference between the controls and PSC group would not have an impact on the results of the study, but obviously this cannot be definitely ruled out.

## 4. Materials and Methods

### 4.1. Subjects

Three groups of subjects were included in the study: (i) PSC patients (n = 10) who had never been diagnosed with concomitant IBD; (ii) PSC-UC patients (n = 10) showing macroscopic features of UC on colonoscopy, which was confirmed with a histology examination, and (iii) UC (n = 10) healthy subjects who underwent colonoscopies for various indications and showed neither macroscopic nor microscopic abnormalities in their colon (controls n = 10). Colon biopsy specimens (for histological and mRNA evaluations) were obtained from the ascending and sigmoid colons from each patient. PBMCs were freshly isolated from heparinised venous blood samples of PSC and PSC-UC patients and healthy subjects.

All included patients with primary sclerosing cholangitis fulfilled EASL criteria for this diagnosis. The ages were between 18 and 75 years.

The main exclusion criteria for the study were: an inability to give informed consent; patients with other forms of chronic liver diseases; a diagnosis of decompensated liver cirrhosis (Child-Pugh class B–C); pregnant or breastfeeding women, and any other condition, which in the opinion of the investigators, would impede the patient’s participation or compliance during the study.

Demographic and clinical data on analysed patients are summarised in Table 1. Written, informed consent was obtained from each patient included in the study. The study protocol was approved by the Ethics Committee of the Pomeranian Medical University (BN-001/43/06 and BN-001/122/06) and adhered to the ethical guidelines of the 1975 Declaration of Helsinki.

### 4.2. RNA and miRNA Expression Analysis

Total RNA was isolated using the RNeasy Mini kit (Qiagen, Hilden, Germany), and cDNA synthesis was carried out using the TaqMan Advanced miRNA cDNA Synthesis Kit (Applied Biosystems, Thermo Fisher Scientific, Waltham, MA, USA) or the SuperScript IV RT (Invitrogen, Carlbad, CA, USA) according to the manufacturer’s protocol. Gene expressions were measured using human TaqMan Gene Expression Assays for 18S ribosomal RNA (Hs99999901_s1), S1PR1 (Hs01922614_m1), SOCS1 (Hs00705164_s1), FOXP3 (Hs01085834_m1), IL-17A (Hs00174383_m1), IL-6 (Hs001741131_m1), STAT3 (Hs00374230_m1), MLH1 (Hs00179866_m1), MSH2 (Hs00954125_m1), MSH6 (Hs00943000_m1).

MiRNA cDNA synthesis was carried out using the TaqMan Advanced miRNA cDNA synthesis kit (Applied Biosystems, Waltham, MA, USA) according to the manufacturer’s protocol. The expression of miR-155 (002623_mir) and reference microRNA (miR-191 (477952_mir) or miR-16 (477860_mir) were measured using TaqMan Advanced miRNA assays and TaqMan Fast Advanced Master Mix (Applied Biosystems, Waltham, MA, USA).

Fluorescence data were analysed using 7500 software v2.0.2. (Applied Biosystems, Waltham, MA, USA) and the relative amounts of transcripts were calculated using the 2-ΔΔCt formula.

### 4.3. PBMCs

Isolation of PBMCs was carried out in accordance with the manufacturer’s protocol (GE Healthcare, Chicago, IL, USA). Changes in miR-155 and SOCS1 mRNA expression were then detected, as described earlier.

### 4.4. ELISA

The PBMCs of phospho-STAT3 [pY705] levels were detected by ELISA (Multispecies ELISA Kit; ThermoFisher Scientific, Carlsbad, CA, USA) according to the manufacturer’s instructions.

### 4.5. Cell Culture, Transfection and Treatments

NCM460D (INCELL Innovative Life Science Solutions, San Antonio, TX, USA; Cell License Material Transfer Agreement #204) is a normal mucosal epithelial cell line, a mixed monolayer/suspension culture, spontaneously immortalized, and non-tumorigenic. Cells were grown in INCELL’s enriched M3:10 BaseF Medium (INCELL Innovative Life Science Solutions San Antonio, TX, USA), 10% *v*/*v* Fetal Bovine Serum (ATCC 30-2025), and 1% antibiotic solution (penicillin/streptomycin; Biowest, Nuaille, France) in accordance with the manufacturer’s recommendations. Caco-2 (HTB-37™) are heterogenous human epithelial colorectal adenocarcinoma cells, and HT-29 (HTB-38™) is a colorectal adenocarcinoma cell line with epithelial morphology. Both were purchased from the American Type Culture Collection and were grown according to the original protocol. All of the cell lines were cultured in 25-cm^2^ or 75-cm^2^ culture flasks and routinely maintained in a humidified atmosphere of 5% CO_2_ at 37 °C.

Transient transfections with either miR-155 inhibitor (Ambion mirVana^®^ Anti-miR miRNA Inhibitor, hsa-miR-155; ID: AM10238; Thermo Fisher Scientific, Waltham, MA, USA) or an miR-155 mimic (Ambion mirVana^®^ miRNA mimic, hsa-miR-155; ID: MC28440; Thermo Fisher Scientific, Waltham, MA, USA) were performed using Lipofectamine RNAiMAX reagent (Invitrogen, Carlsbad, CA, USA) in accordance with the manufacturer’s protocol. Forty-eight hours after transfection, the cells were lysed, and RNA was isolated for further analysis.

To investigate the effect of GCDCA on miR-155 expression, Caco-2 cells were exposed to 200 or 500 µM of GCDCA for 24 h. GCDCA, provided by Sigma (St. Louis, MO, USA), was dissolved in sterile phosphate-buffered saline.

To initiate the inflammatory process, Caco-2, HT-29, and NCM460D cells were incubated in an appropriate complete cell culture medium with the addition of lipopolysaccharides from *Escherichia coli* 0111:B4 (0.5 µg/mL or 1 µg/mL) (4391, Sigma, St. Louis, MO, USA). After 24 h, cells were lysed and RNA was isolated for further analysis.

### 4.6. Statistics

Statistical analyses done with StatView (SAS Institute, Cary, NC, USA), and GraphPad Prism7 applications (GraphPad Software, San Diego, CA, USA). Continuous variables reported as mean + SEM. Statistical differences of baseline characteristics between groups were analyzed using the χ2 test for categorical data, and the Mann-Whitney U test or Student’s *t*-test for quantitative data analysis. Correlation analyses were performed using the Spearman Rank method. Results were considered statistically significant when *p*-values were <0.05.

## 5. Conclusions

**Figure 6:**
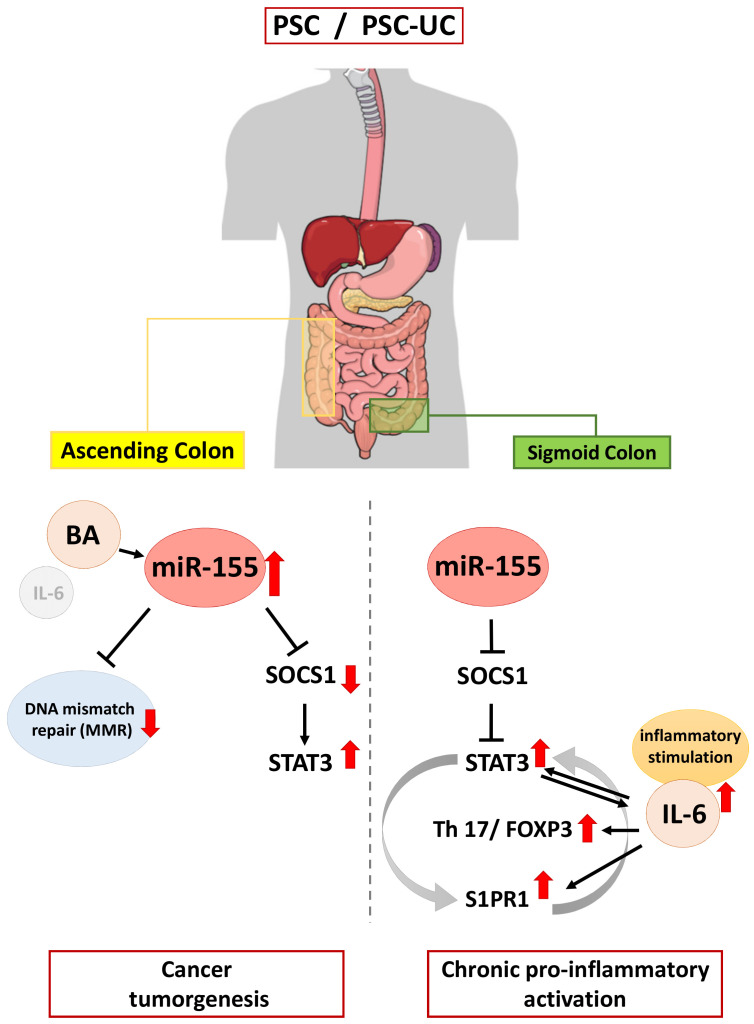
Schematic figure showing the contribution of miR-155 in inflammation and neoplastic transformation in the colon of patients with primary sclerosing cholangitis.

Though great efforts have been made to investigate the pathogenesis of PSC-UC over the past decades, the molecular mechanisms causing it remain largely unknown. A direct comparison of experimental results from an animal model of PSC (mdr2^−/−^ mice develop chronic cholangitis, but not colitis [77]) with the observations from PSC patients is difficult to evaluate [78]. The innovation presented here is the direct study in patients with PSC-UC, in comparison to isolated PSC or UC. This study suggests that miR-155 acts as a link between inflammation and cancer in colitis associated with PSC (Figure 6). The upregulation of miR-155 results in failure of SOCS1 expression and the inhibition of p53, which may predispose patients to pro-tumorigenic transformations. Moreover, the miR-155-modulated suppression of MMR proteins may be responsible for the initiation of colorectal neoplastic transformation in patients with PSC. In contrast, enhanced IL-6/S1PR1 production and a lack of upregulation of miR-155 leads to pro-inflammatory signalling in the sigmoid colon of PSC patients. The observed changes in MMR protein expression, which is representative for MSI-H, could be used for further research focussed on developing useful biomarkers for monitoring colorectal cancer development.

## Figures and Tables

**Table 1:** Demographic and laboratory features of analysed subjects.

	Control (n = 10)	PSC (n = 10)	PSC-UC (n = 10)	UC (n = 10)
**Gender (*Male/Female*)**	6/4	8/2	8/2	2/8
**Age (*years*)**	50 ± 4	28 ± 10	34 ± 15	43 ± 17
**Hb (mg/dl, *normal M: 14–18, F: 12–16*)**	ND	15 ± 0.8	16. ± 1.7	ND
**Bilirubin (mg/dl, *normal < 1.1*)**	ND	1.2 ± 0.4	2.6 ± 0.7	0.4 ± 0.2
**ALP (IU/l, *normal 30–120*)**	ND	304 ± 241	381 ± 277	80 ± 24
**GGTP (IU/l, *normal M < 100, F < 66*)**	ND	325 ± 310	484 ± 232	17 ± 13.3
**ALT (IU/l, *normal < 40*)**	ND	103 ± 72	104 ± 23	15 ± 8.4
**Cirrhosis (*yes/no*)**	N/A	4/6	4/6	N/A

Values are given as mean ± SD, unless stated otherwise. Abbreviations: PSC, primary sclerosing cholangitis; UC, ulcerative colitis; Hb, haemoglobin; ALP, alkaline phosphatase; GGTP, gamma-glutamyl transferase; ALT, alanine aminotransferase; SD, standard deviation; N/A, not applicable; ND, no data.

## Data Availability

Not applicable.
